# Olig2-Induced Neural Stem Cell Differentiation Involves Downregulation of Wnt Signaling and Induction of Dickkopf-1 Expression

**DOI:** 10.1371/journal.pone.0003917

**Published:** 2008-12-18

**Authors:** Sung-Min Ahn, Kyunghee Byun, Deokhoon Kim, Kiyoung Lee, Jong Shin Yoo, Seung U. Kim, Eek-hoon Jho, Richard J. Simpson, Bonghee Lee

**Affiliations:** 1 Center for Genomics and Proteomics, Lee Gil Ya Cancer and Diabetes Institute, Gachon University of Medicine and Science, Incheon, Korea; 2 Mass Spectrometry Analysis Group, Korea Basic Science Institute, Daejeon, Korea; 3 Gachon Institute for Regenerative Medicine, Gachon University of Medicine and Science, Incheon, Korea; 4 Department of Medicine, University of British Columbia, Vancouver, Canada; 5 Department of Life Science, The University of Seoul, Dongdaemun-gu, Seoul, Korea; 6 Joint Proteomics Laboratory, Ludwig Institute for Cancer Research & the Walter and Eliza Hall Institute of Medical Research, Melbourne, Australia; University of Washington, United States of America

## Abstract

Understanding stem cell-differentiation at the molecular level is important for clinical applications of stem cells and for finding new therapeutic approaches in the context of cancer stem cells. To investigate genome-wide changes involved in differentiation, we have used immortalized neural stem cell (NSC) line (HB1.F3) and Olig2-induced NSC differentiation model (F3.Olig2). Using microarray analysis, we revealed that Olig2-induced NSC differentiation involves downregulation of Wnt pathway, which was further confirmed by TOPflash/FOPflash reporter assay, RT-PCR analysis, immunoblots, and immunocytochemistry. Furthermore, we found that Olig2-induced differentiation induces the expression of Dickkopf-1(Dkk1), a potent antagonist of Wnt signaling. Dkk1 treatment blocked Wnt signaling in HB1.F3 in a dosage-dependent manner, and induced differentiation into astrocytes, oligodendrocytes, and neurons. Our results support cancer stem cell hypothesis which implies that signaling pathway for self-renewal and proliferation of stem cells is maintained till the late stage of differentiation. In our proposed model, Dkk1 may play an important role in downregulating self-renewal and proliferation pathway of stem cells at the late stage of differentiation, and its failure may lead to carcinogenesis.

## Introduction

It has been widely accepted until recently that no new neurons are generated after neurogenesis is completed during the early embryonic development (i.e., there are no resident stem cells in the nervous system) [1]. More recent studies, however, led to the isolation of neural stem cells (NSCs) from the embryonic mammalian central nervous system (CNS) [2]–[4], followed by the isolation of NSCs from the adult mammalian CNS [5], [6]. These discoveries revealed the regenerative power of the CNS, which may be used for therapeutic purposes [7].

Currently, there are four main strategies in NSCs and their progenitor cell-based therapy: transplantation of oligodendrocyte progenitor cells for treating myelin disorders; transplantation of neuronal progenitor cells to treat diseases of discrete loss of a single neuronal phenotype, such as Parkinson disease; implantation of mixed progenitor pools to treat diseases resulting from the loss of several phenotypes, such as spinal cord injury; mobilization of endogenous neural progenitor cells to treat neurodegenerative diseases [8]. Despite significant progress that has been made for clinical application of NSCs, key questions about global perspectives for the differentiation pathway remain to be answered including molecular determinants of neural and glial fates and distinctive stages of differentiation [9].

Understanding differentiation is important for at least two reasons. Firstly, differentiation is a process of acquiring specific functions of committed cells. Therefore, understanding each step of differentiation, and characterizing differentiation phenotypes are the basis of stem cell engineering. Future stem cell research is likely to focus on improving the ability to guide the differentiation of stem cells and to control their survival and proliferation for clinical application [10]. Secondly, understanding differentiation may provide an important clue for treating cancers. According to the newly emerging cancer stem cell hypothesis, tumors seem to arise from small populations of cancer stem cells that originate from the transformation of normal stem cells [11]. In this hypothesis, a tumor can be viewed as an aberrant organ initiated by a cancer stem cell that undergoes processes analogous to the self-renewal and differentiation of normal stem cells [12]. Although similar to normal stem cells in many ways, cancer stems cells are critically different in that their transit-amplifying progeny do not mature and die as do the progeny of normal stem cells (maturation arrest) [13]. Therefore, understanding differentiation may ultimately lead to the development of differentiation therapy, which is directed toward reversal of the maturation arrest, thus allowing the cancer cells to differentiate and die eventually [14].

**Figure 1:**
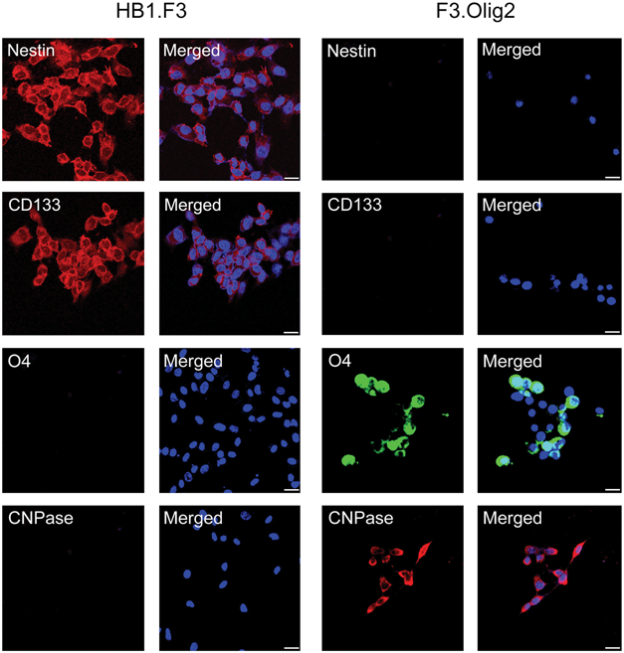
Expression of lineage-specific markers in HB1.F3 and F3.Olig2. Neural stem cell markers such as nestin [52] and CD133 [53] are expressed only in HB1.F3 wherease oligodendrocytes markers such as O4 [54] and CNPase [55] are expressed only in F3.Olig2. Merged; markers with DAPI, Bar = 50 µm

To identify genes and pathways that could play a role in the differentiation of NSCs, we performed microarray analysis using immortalized neural stem cell line (HB1.F3) and its oligodendrocyte progeny (F3.Olig2) in which olig2 is over-expressed. It has been shown that olig2 overexpression can induce the in vitro differentiation of NSCs into mature oligodendrocytes [15]. HB1.F3 has the ability to self-renew and differentiate into cells of neuronal and glial lineages in both in vivo and in vitro [16], [17]. F3.Olig2 cells express oligodendrocyte markers and represent a model of NSC differentiation (Fig. 1).

## Results

### Downregulation of Wnt pathway in F3.Olig2

**Figure 2:**
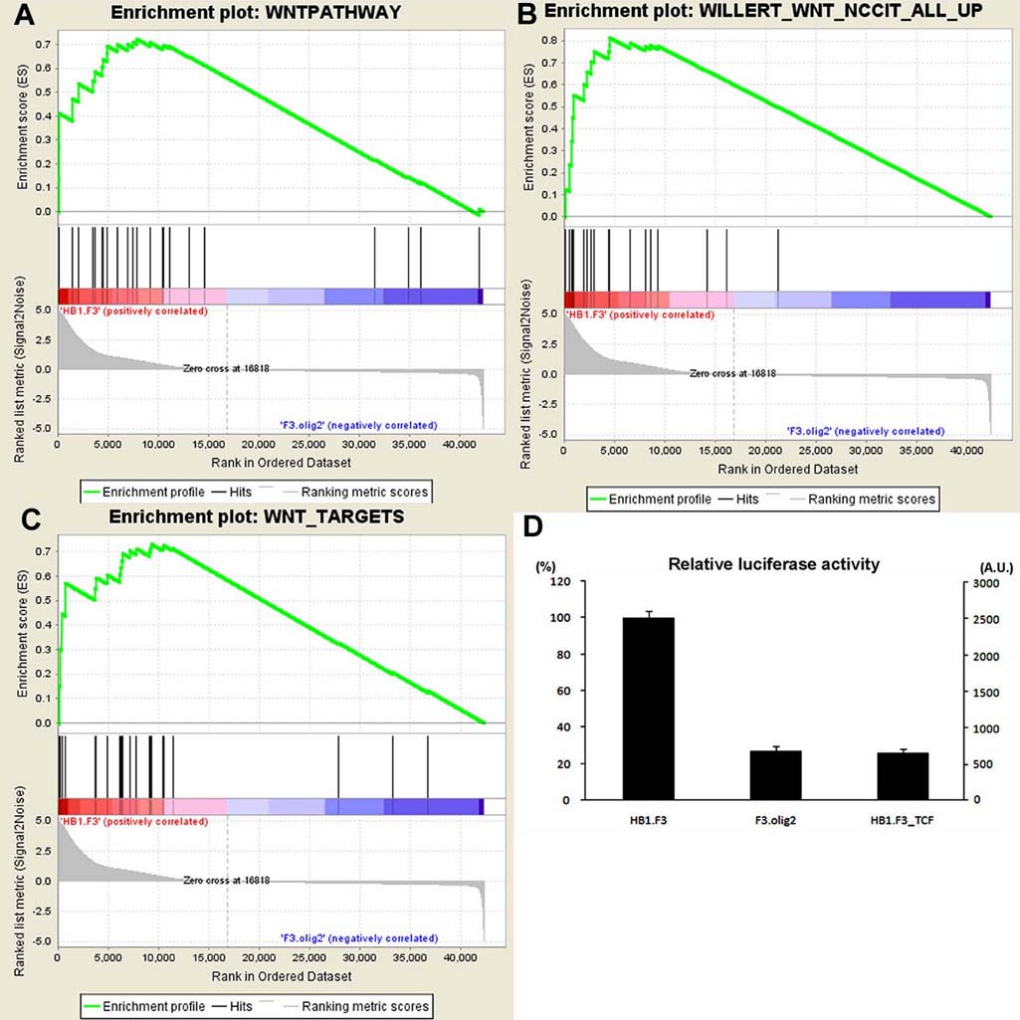
Downregulation of Wnt pathway in F3.Olig2. GSEA shows that Wnt pathway genes (A), Wnt-responsive genes (B), and Wnt pathway target genes (C) are enriched in HB1.F3. TOPFLASH/FOPflash reporter analysis (D) confirms that Wnt pathway activity in HB1.F3 is significantly higher than that in F3.Olig2. The reporter activity in HB1.F3 was inhibited to the level of F3.Olig2 when HB1.F3 was co-transfected with ‘dominant negative’ (DN) TCF constructs. Data are means±S.D. of triplicate samples. A.U., Activity unit

Microarray analysis revealed global gene expression changes between HB1.F3 and F3.Olig2; more than 60% of genes that are present in HB1.F3 are absent in F3.Olig2. Since the global gene expression changes violate basic assumptions of statistical analysis of microarray data that most genes are not differentially expressed [18], we have employed the knowledge-based Gene Set Enrichment Analysis (GSEA) (Materials and Methods), instead of using conventional statistical analysis such as t-test, to investigate expression changes in functional groups of genes. Since the Wnt pathway is known to be involved in neural stem cell-differentiation in contra-acting ways (i.e., maintain stemness versus inducing differentiation [19]–[21], the investigation of the microarray data was focused on Wnt pathway-related gene sets. Using this method, we identified significant enrichment of Wnt pathway genes, genes upregulated by Wnt [22], and Wnt pathway target genes in HB1.F3, an immortalized neural stem cell line (Fig. 2A–C) (see Table S1, S2, S3 for detailed information). To obtain further evidence that Wnt pathway is active in HB1.F3 and suppressed in F3.Olig2, we transfected a transcription factor (TCF) reporter gene (TOPflash) containing five optimal TCF-binding sites or the mutant control plasmid (FOPflash) into HB1.F3 and F3.Olig2. pRL-TK was included to normalize data for transfection efficiency [23]. As shown in Fig. 2D, the reporter activity of Wnt pathway is more than three times higher in HB1.F3 than in F3.Olig2. The addition of dominant negative TCF plasmids (dnTCF) decreased the reporter activity in HB1.F3 close to the level of F3.Olig2, indicating that the Wnt activity in F3.Olig2 is at the background level.

### Validation of gene expression using semiquantitative RT-PCR

**Figure 3:**
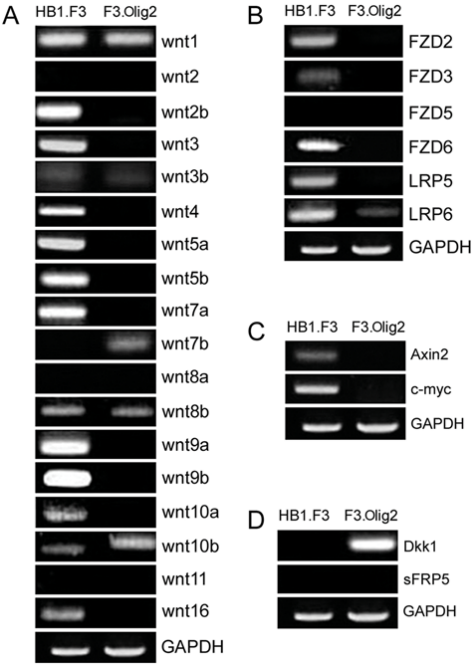
RT-PCR analysis of Wnt pathway-related genes. A. Most Wnt genes are overexpressed in HB1.F3 whereas the expression of Wnt 10b is increased in F3.Olig2. Interestingly, Wnt7b is expressed only in F3.Olig2. B. Except FZD5, all Wnt receptor and co-receptor genes that are expressed in HB1.F3 are suppressed in F3.Olig2. C. Wnt pathway target genes are expressed only in HB1.F3. D. Dkk1, a soluble Wnt antagonist, is expressed only in F3.Olig2.

As described above, both GSEA and the reporter assay revealed that Wnt pathway is active in HB1.F3 and downregulated in F3.Olig2. To further confirm these findings at the molecular level, we performed semiquantitative RT-PCR analysis of Wnt pathway components including 18 Wnt families, 4 Wnt receptors, 2 Wnt co-receptors, 2 antagonists of Wnt signaling, and 2 target genes of Wnt pathway. Most of Wnt genes which expressed in HB1.F3 are suppressed in F3.Olig2, whereas only Wnt 10b gene is increased in F3.Olig2 (Fig. 3A). Interestingly, Wnt7b is expressed only in F3.Olig2. Wnt receptors and co-receptor are all downregulated in F3.Olig2 and frizzled 5(FZD5) was not expressed in both HB1.F3 and F3.Olig2 (Fig. 3B). In accordance with GSEA results, Wnt pathway target genes such as Axin2 [24] and c-myc [25] are expressed only in HB1.F3 (Fig. 3C). On the other hand, Dickkopf-1 (Dkk1), a secreted Wnt antagonist [26], is expressed only in F3.Olig2 (Fig. 3D). Unlike other Wnt antagonists, the function of Dkk1 is independent of Frizzled, and inhibits canonical Wnt signaling by binding to LRP6 [27], [28], the only Wnt co-receptor expressed in F3.Olig2.

### Levels of β-catenin and phosphor-β-catenin (p-β-catenin), and their subcellular localization

**Figure 4:**
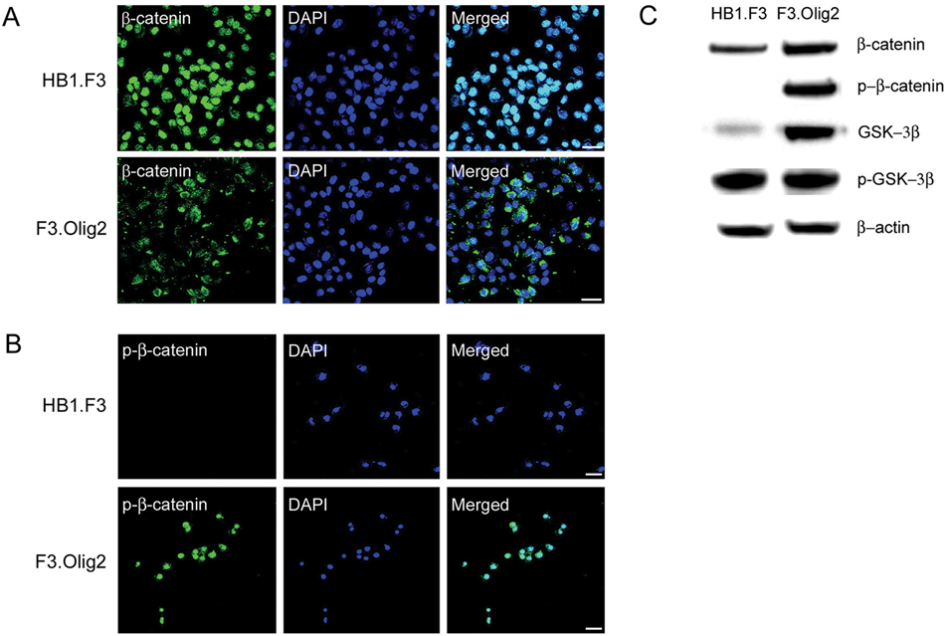
Levels of β-catenin and phospho-β-catenin (p-β-catenin), and their subcellular localization. In HB1.F3, β-catenin is mainly localized in nucleus (A), and p-β-catenin is not detected (B, C). In F3.Olig2, β-catenin is mainly localized in cytoplasm (A), and p-β-catenin is detected and mainly localized in nucleus (B, C). The level of GSK3β, which phosphorylates β-catenin on Ser-33/Ser-37/Thr-41, is increased in F3.Olig2 (C). Bar = 50 µm

Wnt signaling is transduced to β-catenin in cytoplasm, which enters the nucleus and activate transcription of Wnt pathway target genes with TCF [29]. On the other hand, phosphorylation of β-catenin leads to ubiquitination and degradation of β-catenin [30], [31]. In HB1.F3, β-catenin is mainly localized in nucleus and p- β-catenin (pS_33_/pS_37_/pT_41)_ is not detected (Fig. 4A, B). In F3.Olig2, β-catenin is mainly localized in cytoplasm, and p- β-catenin pS_33_/pS_37_/pT_41)_ is exclusively observed in nucleus (Fig. 4A, B). In immunoblot analysis (Fig. 4C), the expression of β-catenin is increased in F3.Olig2 and p- β-catenin (pS_33_/pS_37_/pT_41)_ was detected only in F3.Olig2. The level of GSK3β, which phosphorylates β-catenin on S33/S37/T41 [32], is also increased in F3.Olig2.

### Suppression of Wnt signaling and induction of differentiation by Dkk1

**Figure 5:**
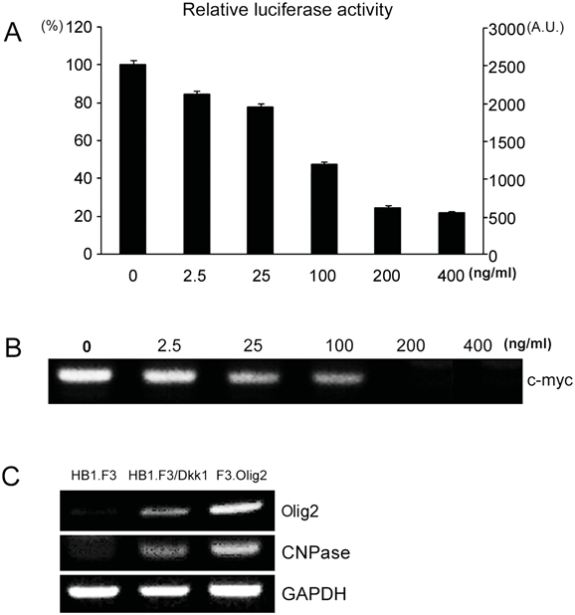
Dkk1 inhibits Wnt signaling in HB1.F3. A. TOPflash/FOPflash reporter assay shows that Dkk1 represses Wnt signaling in HB1.F3 in a dosage-dependent manner. Data are means±S.D. of triplicate samples. B. Dkk1 represses the expression of c-myc, one of the target genes of Wnt pathway, in HB1.F3 in a dosage-dependent manner. C. Dkk1 treatment induces the expression of oligodendrocytes markers (Olig2 and CNPase) in HB1.F3.

**Figure 6:**
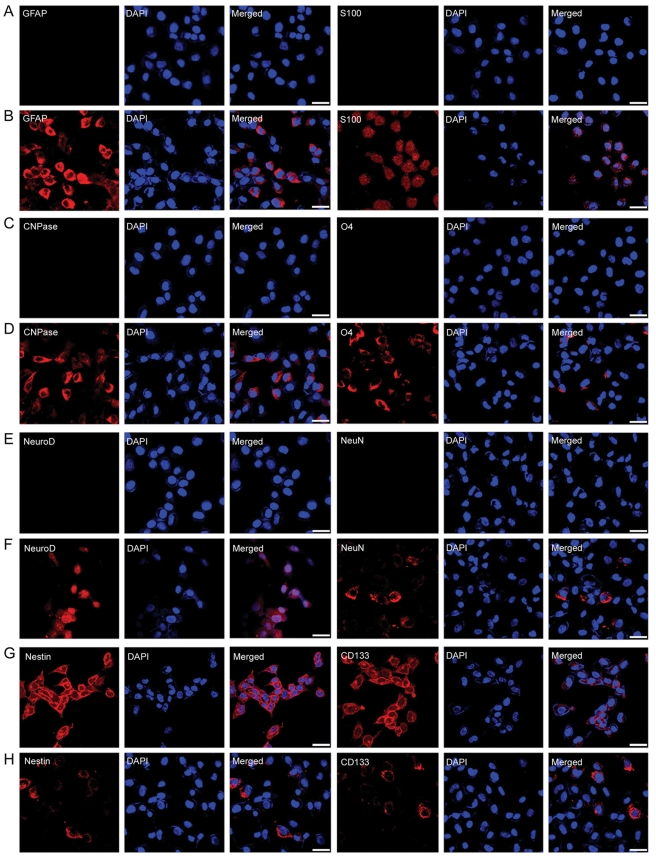
Immunohistochemical staining of HB1.F3 or HB1.F3 with Dkk1 treatment for various markers of stem cell and differentiated cells. Dkk1 induces differentiation of HB1.F3 into astrocytes, neurons and oligodendrocytes. Astrocyte markers (GFAP, S100), oligodendrocyte markers (CNPase, O4), neuron markers (NeuroD, NeuN) and neural stem cell markers(Nestin, CD133) were double stained with DAPI (blue) in HB1.F3 before (A, C, E, G ) and after (B, D, F, H ) Dkk1 treatment. Bar = 50 µm

As shown in Fig. 3D, Dkk1, a potent antagonist of Wnt signaling, is expressed only in F3.Olig2. We also tested the effect of Dkk1 on HB1.F3. When HB1.F3 cells were treated with Dkk1, Wnt signaling in HB1.F3 was inhibited in a dosage-dependent manner (Fig. 5A). Dkk1 treatment decreased the expression of c-myc, a Wnt pathway target gene, in a dosage-dependent manner (Fig. 5B). Dkk1 treatment also induced the expression of oligodendrocyte markers such as Olig2 and CNPase in HB1.F3 (Fig. 5C). Furthermore, Dkk1 treatment induced differentiation of HB1.F3 into astrocytes, neurons, and oligodendrocytes (Fig. 6, Fig. S1). As for differentiation efficiency, astrocytes were the highest, oligodendrocytes the second, and neurons the lowest.

## Discussion

In the present study, we showed that Olig2-induced differentiation of NSCs leads to downregulation of Wnt pathway, which is known to regulate the balance between self-renewal and differentiation in CNS [33]. Although Wnt signaling can influence cell lineage decisions such as neural differentiation of NSCs [19], differentiation of embryonic stem cells into dorsal interneurons [34], and differentiation of NSCs into dopaminergic neurons [21], Wnt signaling predominates in stem cell proliferation and neural stem cell expansion [20], and inhibits differentiation [21], [35].

Unlike these studies that focused on modulating Wnt signaling and analyzing its effects on stem cells, we found, in the present study, that the differentiation-inducing event (*i.e*., overexpression of Olig2) may precede the downregulation of Wnt pathway.

We found that most of genes, receptors, co-receptors, and target genes expressions were increased in HB1.F3, but that of Wnt inhibitor was increased in F3.Olig2. And β-catenin was observed in cytoplasm of F3.Olig2 and nucleus of HB1.F3, whereas p-β-catenin was expressed only in the nucleus of F3.Olig2. This means canonical Wnt pathway may be activated in HB1.F3, and decreased in F3.Olig2.

Furthermore, we showed that the expression of Dkk1, a potent antagonist of Wnt signaling, is induced in F3.Olig2. Dkk1 treatment blocks Wnt signaling in HB1.F3, and induces differentiation into astrocytes, oligodendrocytes, and neurons. These findings comply with previous findings that blocking Wnt pathway induces differentiation [21], but not lineage-specific. It is generally accepted that there is the balance between self-renewal and differentiation [33], which may be manifested in two different ways. When Olig2, a differentiation-inducing signal, was overexpressed, this led to lineage-specific differentiation of neural stem cells and downregulation of Wnt pathway (*i.e*., self-renewal pathway) as demonstrated by F3.Olig2. When HB1.F3 cells were treated with Dkk1, a Wnt inhibitor, this led to downregulation of Wnt pathway and lineage-non-specific differentiation.

According to previous findings, Dkk1 is a direct target of the β-catenin/TCF transcription complex that mediates Wnt signaling [36]–[38]. Although these studies indicate that Dkk1 forms a novel feedback loop in Wnt signaling, our results suggest that the expression of Dkk1 is induced by a different pathway in F3.Olig2 since Wnt signaling as well as Wnt genes and receptors are suppressed in F3.Olig2. Previous reports showed that the expression of Dkk1 can be induced, independent of Wnt signaling, by differentiation-promoting reagents such as 1α, 25-dihydroxyvitamin D3 [39] and retinoic acids [40]. Dkk1 can be also induced by p53 [41].

Evidences from our experiments provide a probable link between stem cell maturation arrest and carcinogenesis at the molecular level. According to cancer stem cell hypothesis, tumors arise from maturation arrest of stem cells [42], which implies that signaling pathway for self-renewal and proliferation of stem cells is maintained till the late stage of differentiation. In our proposed model (Fig. 6), Wnt signaling, which is important for self-renewal and proliferation of NSCs, is turned off at the late stage of differentiation by Dkk1, which is turned on not by Wnt pathway but by a differentiation-related pathway. The feasibility of this model is supported by experimental evidences that Dkk1 is epigenetically silenced in many tumors including gastrointestinal tumors [43], [44], cervical cancers [45], leukemia [46], and medulloblastoma [47]. Also, in HeLa cells, Dkk1 is required for tumorigenicity [48]. Altogether, these evidences may indicate that Dkk1 play an important role in downregulating self-renewal and proliferation pathway of stem cells at the late stage of differentiation, and its failure may lead to carcinogenesis.

## Materials and Methods

### Cell lines

Stable clonal human NSC line, HB1.F3, was generated by retroviral transduction of primary fetal human neural stem cells (hNSCs) with an avian v-*myc* cell cycle regulatory gene as previously reported (Kim et al, 2008; Production and characterization of immortal human neural stem cell line with multipotent differentiation property [49]. F3.Olig2 was generated by overexpressing Olig2 in HB1.F3. Briefly, Olig2 cDNA was ligated into multiple cloning sites of the retroviral vector pLHCX (Clontech, Mountain View, CA). PA317 amphotropic packaging cell line was infected with the recombinant retroviral vector, and the supernatants from the packagaing cells were added to the HB1.F3 cells. Stably transfected colonies were selected by hygromycin resistance (Kim *et al*., in submission).

### Cell Culture

HB1.F3 and F3.Olig2 cells were maintained in Dulbecco's modified Eagles medium (DMEM) supplemented with 5% fetal bovine serum (FBS), 5% horse serum, and 50 µg/ml gentamycin at 37°C and 5% CO_2_. Dkk1 was acquired from R&D system (1096-DK) and added to HB1.F3 cells at a concentration from 25 ng/mL to 400 ng/mL. Dkk1-treated HB1.F3 cells were harvested 6 hrs after treatment.

### Microarray analysis

Five replicates of each cell line were used for isolating and purifying RNA by Qiagen RNeasy kit. The resulting total RNA samples were further assessed for integrity prior to chipping using BioRad Experion System. Microarray experiments were performed using Sentrix Human-6 Whole Genome Expression BeadChips (Illumina), analyzing over 46,000 known genes, gene candidates, and splice variants according to manufacturer's instructions.

### Bioinformatics

Microarray data were normalized using median normalization method. Pathway analysis of the gene expression data was performed using the Gene Set Enrichment Analysis (GSEA), which is able to detect coordinate changes in the expression of groups of functionally related genes [50], [51]. An enrichment score (ES) was calculated for each gene set in GSEA and the statistical significance of the ES was estimated by an empirical permutation test using 1,000 gene permutations to obtain the nominal p-value. GSEA software was downloaded from http://www.broad.mit.edu/gsea/

### Luciferase reporter assay

Cells were transiently transfected using the basic nucleofector kit for primary mammalian neural cells (Amaxa biosystems) according to the manufacturer's instructions. Briefly, after trypsinization, cell counting using hemocytometer, and centrifugation, ∼5×10^6^ cells were re-suspended in 100 µl of Basic Nucleofector Solution (Amaxa biosystems), and transfected with 1 µg of reporter plasmids (pTOPflash or pFOPflash), and 1 µg of internal control pRL-TK; with or without 4 µg of dominant negative (DN)-TCF plasmids (program A33). After transfection, ∼1×10^4^ cells were transferred to each well of a 24-well plate containing fresh, pre-warmed DMEM and maintained for 48 hrs at 37°C and 5% CO_2_. Then, the cells were lysed in lysis buffer, and 20 µl of each lysate was monitored for luciferase activity using Dual-Luciferase Reporter Assay System (Promega). Light units were measured using Lmax II 384 (Molecular devices).

A control reporter, pRL-TK contains a herpes simplex virus thymidine kinase promoter driving a Renilla luciferase gene, and Renilla luciferase activity was used to normalize the results for transfection efficiency. The reporter activities were shown as the ratios of TOPflash to FOPflash luciferase activity from triplicate experiments.

### RT-PCR

RNA was isolated from six biological replicates from each group using Qiagen RNeasy MiniKit (Qiagen), pooled, and subjected to first-strand cDNA synthesis using Reverse Transcription System (Promega, A3500) according to the manufacturer's instructions. Amplification was carried out at 94°C for 2 min, followed by 30 cycles at 94°C for 1 min, at appropriate annealing temperatures for each primer for 1 min, and at 72°C for 1 min 30 s. Sequences of primers used for RT-PCR are summarized in Supplementary Table S1.

### Immunoblot

Cell lysates were prepared with lysis buffer containing 7 M urea, 2 M thiourea, and 4% CHAPS. Equal amounts (25 µg) of protein from each group were separated in 4–12% polyacrylamide gels (Invitrogen) and transferred to nitrocellulose membrane (Millipore). The primary antibodies used were anti-β-catenin (1∶1000, #9562), anti-phospho-pS_33_/pS_37_/pT_41_-β-catenin (1∶1000, #9561), anti-GSK3 (1∶1000, #9315), anti-p-GSK3β (1∶1000, #9336), and anti-β-actin (1∶1,000, #4967 ) from Cell Signaling Technology.

### Immunofluorescent analysis

Cells were grown on Lab-Tek II chamber slide (Nalge Nunc Int., Naperville, IL), rinsed in PBS, fixed in 4% paraformaldehyde for 20 min, and rinsed again in PBS. The cells were incubated for overnight at 4°C with following antibodies: β-catenin antibody (1∶200, Cell signaling, #9562); p-β-catenin antibody (1∶200, Cell signaling, #9561); Nestin antibody (1∶100, Chemicon, MAB5326); CD133 antibody (1∶100, abcam, ab161518); O4 antibody (1∶200, Chemicon, MAB345); CNPase antibody (1∶200, Chemicon, MAB326); GFAP antibody (1∶1,000, Chemicon, AB9598); S100 antibody ( 1∶1,000, Kamiya biomedical company, MC-032). The cells were rinsed in PBS and incubated for 1 h at room temperature with Alexa fluor 594 goat anti rabbit IgG (1∶500, molecular probes Inc., A11012); Alexa fluor 594 goat anti mouse IgG (1∶500, molecular probes Inc., A11005); Alexa fluor 488 rabbit anti goat IgG (1∶500, molecular probes Inc., A11078); Alexa fluor 488 donkey anti rabbit IgG (1∶500, molecular probes Inc., A21206). For counterstaining of cell nuclei cells were incubated with DAPI (4′6-diamidino-2-phenylindole; 1 µg/ml, Sigma-Aldrich) in H_2_O for 40 sec. After wash in PBS, coverslips were mounted onto glass slides using Fluoroguard Antifade reagent (Bio-Rad Laboratories, Hercules, CA), and examined under a laser confocal fluorescence microscope (FV500, Olympus, Japan).

## Supporting Information

Figure S1 Phase contrast(DIC) and merged images of the immunohistochemical staining of HB1.F3 or HB1.F3 with Dkk1 treatment for various markers of stem cell and differentiated cells. Merged images of DAPI and astrocyte markers (GFAP, S100), oligodendrocyte markers (CNPase, O4), neuron markers (NeuroD, NeuN) and neural stem cell markers(Nestin, CD133) were compared with DIC images in HB1.F3 before( A, C, E, G ) and after( B, D, F, H ) Dkk1 treatment. Bar = 50 µm
(10.49 MB TIF)

Table S1 GSEA details of Wnt pathway genes
(0.06 MB DOC)

Table S2 GSEA details of Wnt-responsive genes
(0.05 MB DOC)

Table S3 GSEA details of Wnt-responsive genes
(0.05 MB DOC)
