# Supplementary figures and images for: Olig2-Induced Neural Stem Cell Differentiation Involves Downregulation of Wnt Signaling and Induction of Dickkopf-1 Expression
Source: PLoS One. 2008 Dec 18;3(12):e3917. doi: 10.1371/journal.pone.0003917 (PMC2602983; doi:10.1371/journal.pone.0003917)

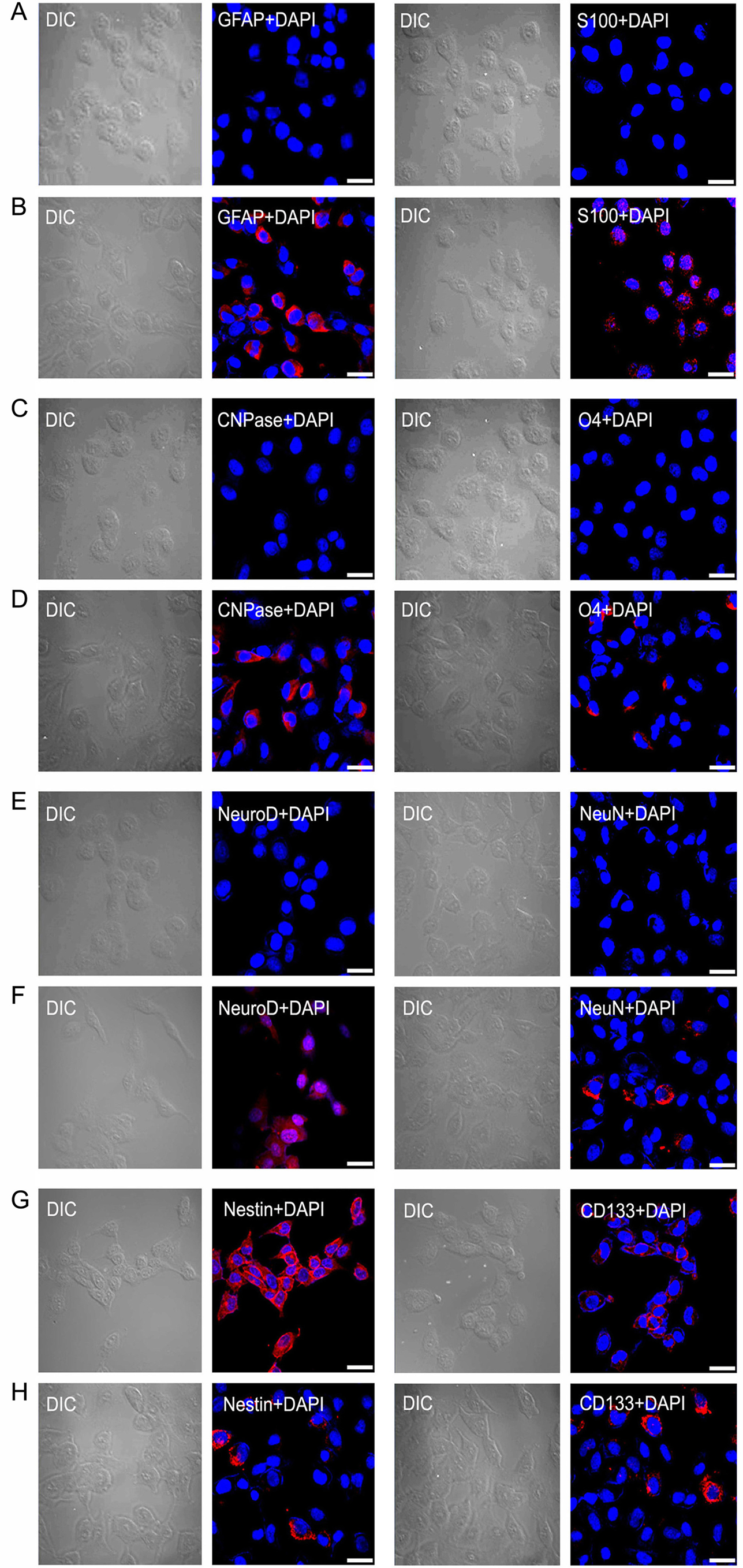

Supplement: Figure S1 — Phase contrast(DIC) and merged images of the immunohistochemical staining of HB1.F3 or HB1.F3 with Dkk1 treatment for various markers of stem cell and differentiated cells. Merged images of DAPI and astrocyte markers (GFAP, S100), oligodendrocyte markers (CNPase, O4), neuron markers (NeuroD, NeuN) and neural stem cell markers(Nestin, CD133) were compared with DIC images in HB1.F3 before( A, C, E, G ) and after( B, D, F, H ) Dkk1 treatment. Bar = 50 µm (10.49 MB TIF) [file pone.0003917.s001.tif]
