# Supplementary material for: Olig2-Induced Neural Stem Cell Differentiation Involves Downregulation of Wnt Signaling and Induction of Dickkopf-1 Expression
Source: PLoS One. 2008 Dec 18;3(12):e3917. doi: 10.1371/journal.pone.0003917 (PMC2602983; doi:10.1371/journal.pone.0003917)
Supplement: Table S1 — GSEA details of Wnt pathway genes (0.06 MB DOC) [file pone.0003917.s002.doc]

| **GENE SYMBOL** | **RANK IN GENE LIST** | **RANK METRIC SCORE** | **RUNNING ES** | **CORE ENRICHMENT** |
| --- | --- | --- | --- | --- |
| HDAC1 | 91 | 4.918485641 | 0.13626745 | Yes |
| CCND1 | 122 | 4.893132687 | 0.2732643 | Yes |
| PPP2CA | 127 | 4.884833813 | 0.41064262 | Yes |
| GSK3B | 1473 | 3.302010298 | 0.4717557 | Yes |
| CSNK1D | 2044 | 2.702620029 | 0.53433216 | Yes |
| MAP3K7 | 3451 | 1.650765777 | 0.5475317 | Yes |
| CSNK2A1 | 3696 | 1.522304773 | 0.58460206 | Yes |
| TLE1 | 4368 | 1.282360315 | 0.60481936 | Yes |
| CTBP1 | 4514 | 1.242798686 | 0.63636535 | Yes |
| MAP3K7IP1 | 4873 | 1.172144651 | 0.6608847 | Yes |
| NLK | 4919 | 1.163506031 | 0.69256455 | Yes |
| FZD1 | 5885 | 1.029806256 | 0.69872 | Yes |
| MYC | 6914 | 0.902459323 | 0.6998013 | Yes |
| CSNK1A1 | 7496 | 0.823045135 | 0.7092211 | Yes |
| AXIN1 | 7891 | 0.765806317 | 0.7214533 | Yes |
| BTRC | 9157 | 0.570062339 | 0.70757407 | No |
| PPARD | 10388 | 0.383456081 | 0.6892711 | No |
| FRAT1 | 10525 | 0.363739312 | 0.6962908 | No |
| CREBBP | 11135 | 0.279207587 | 0.6897431 | No |
| APC | 13019 | 0.115453772 | 0.6484517 | No |
| DVL1 | 14599 | 0.054819606 | 0.6126447 | No |
| Tcf1 | 31508 | -0.190651581 | 0.21806692 | No |
| WNT1 | 34836 | -0.231948346 | 0.14589745 | No |
| WIF1 | 36061 | -0.250430465 | 0.123992674 | No |
| CTNNB1 | 41932 | -0.837212682 | 0.008704728 | No |

ES represent enrichment score.
