# Supplementary material for: Olig2-Induced Neural Stem Cell Differentiation Involves Downregulation of Wnt Signaling and Induction of Dickkopf-1 Expression
Source: PLoS One. 2008 Dec 18;3(12):e3917. doi: 10.1371/journal.pone.0003917 (PMC2602983; doi:10.1371/journal.pone.0003917)
Supplement: Table S2 — GSEA details of Wnt-responsive genes (0.05 MB DOC) [file pone.0003917.s003.doc]

|  | |  | | | |
| --- | --- | --- | --- | --- | --- |
| **GENE SYMBOL** | **RANK IN GENE LIST** | | **RANK METRIC SCORE** | **RUNNING ES** | **CORE ENRICHMENT** |
| CCND1 | 122 | | 4.893132687 | 0.12528862 | Yes |
| SRP19 | 525 | | 4.553001881 | 0.23504542 | Yes |
| SNRPA1 | 722 | | 4.312948227 | 0.34338614 | Yes |
| SLC2A1 | 815 | | 4.172191143 | 0.45049948 | Yes |
| NCOA3 | 930 | | 4.000826359 | 0.5526036 | Yes |
| ING1 | 1953 | | 2.784901142 | 0.60138214 | Yes |
| GSTM3 | 2291 | | 2.487021685 | 0.65855855 | Yes |
| NSMAF | 2688 | | 2.144352913 | 0.7053635 | Yes |
| UBE2D2 | 2934 | | 1.966875672 | 0.75109076 | Yes |
| TLE1 | 4368 | | 1.282360315 | 0.7507903 | Yes |
| GJA1 | 4463 | | 1.25673008 | 0.78148675 | Yes |
| MSX2 | 4493 | | 1.248995185 | 0.81351787 | Yes |
| CITED2 | 6579 | | 0.945048988 | 0.78896135 | No |
| EIF3S1 | 8012 | | 0.749424219 | 0.7747244 | No |
| GYPC | 8575 | | 0.6679492 | 0.7789295 | No |
| ID2 | 9239 | | 0.557241917 | 0.7778458 | No |
| TNFRSF11B | 14188 | | 0.0674472 | 0.6625888 | No |
| EDNRB | 16123 | | 0.013931978 | 0.6172132 | No |
| CSTF1 | 21239 | | -0.071322106 | 0.498108 | No |

ES represent enrichment score.
